# Confirmation bias and quantitative approach in psychiatry: should ideological competing interests be declared?

**DOI:** 10.3389/fpsyt.2024.1365733

**Published:** 2024-04-24

**Authors:** Martin Blay, Christophe Gauld, Pauline Espi, Bruno Falissard

**Affiliations:** ^1^ADDIPSY, Outpatient Addictology and Psychiatry Center, Santé Basque Développement Group, Lyon, France; ^2^Université Paris-Saclay, Université de Versailles Saint-Quentin-en-Yvelines (UVSQ), INSERM, Centre de Recherche en Epidémiologie et Santé des Populations Team “DevPsy”, Villejuif, France; ^3^Hôpital Femme-Mère-Enfant, Hospices Civils de Lyon, Lyon, France

**Keywords:** quantitative approach, qualitative approach, ideology, confirmation bias, epistemology

## 1 Introduction

Humans have always sought to find the most accurate way to approach and understand reality. This assumption is particularly true in medical sciences, where two visions of knowledge have been opposed: a quantitative, rational, mathematical vision, embodied by fundamental sciences; and a spiritual, qualitative, philosophical vision, embodied by human sciences (1). This opposition mirrors the foundational sociological divide between Durkheim’s holism (“the whole explains the parts”) and Weber’s methodological individualism (“the parts explain the whole”) (2): quantitative approach may be compared to holism (by suggesting that knowledge should rely on evidence gathered from large groups), whereas qualitative approach may be compared to individualism (by suggesting that the observation of some individuals can help to build knowledge on the overall group). This opposition also has its corollary in the fields of psychology and psychiatry, especially in the second half of the 20^th^ century. On one side, the development and advent of the psychoanalytic theory of mental disorders in the first half of the century can be seen as an example of a rather qualitative understanding of mental disorders. On the other side, the second half of the century saw the development of a more quantitative approach of mental disorders, well represented by RCT-based psychopharmacology and quantitative psychology. Even though these two dominant approaches were not the only available (as emphasized, for instance, by the large number of innovations in the field of social psychiatry in 1960-1970), and even though this binary distinction can be considered as oversimplifying, this opposition between proponents of quantitative psychiatry (arguing for a more objective approach of mental health disorders) and advocates of a more qualitative psychiatry (seen as being more holistic and closer to human intimacy) has led to large debates that continue to unfold within research and clinical communities.

Confirmation bias can be defined as the tendency to conduct research, interpret data or recall information in a way that systematically impacts the possibility that the hypothesis is false, while neglecting information that could challenge or contradict that hypothesis (3). Such pitfalls have been described for qualitative approach of psychiatry, for which psychoanalysis can be used as an example (4). Moreover, many experts have also underlined the risk of such bias in quantitative medical research (e.g. (5–8)), and the same is true for our fields (i.e., psychology and psychiatry). In his popular manifesto on the importance of reforming quantitative psychology, Chris Chambers (9) asks one of the most important questions concerning this field: “Are research psychologists just poorly paid lawyers?”. Indeed, quantitative research in psychology has been the subject of epistemological and methodological critics in the last decades. Alongside the lack of reproducibility and reliability, and the pressure for publications and citations attached to the academic world, Chambers described how, whether consciously or unconsciously, the researcher can bend the way in which statistical analyses are conducted, according to his or her preconceived opinions and/or what he or she needs the results to be. These concerns regarding the non-consideration of the potential influence of scientists’ perspectives on their results are also shared among methodologists (10). In our opinion, beyond the various academic warranties brought up by Chambers, it seems legitimate to also ask the question of the influence of the context in which the project, the analysis of results, or their interpretation is conducted.

Indeed, epistemology has told us that science, as a human practice, is profoundly influenced by the historical, sociological, economic and political context in which it is practiced (11). Because of the social utility it can have (in the sense that it can influence decision-making, whether medical or political), research can be subject to confirmation bias when its priorities are aligned with the social utility one wishes to confer to it (7). This consideration is also shared among epistemologists working on confirmation bias: while some experts see it as a pernicious tendency, others consider it adaptive, notably because of its usefulness to influence people and social structures to make them match our beliefs about them (12). This may be particularly relevant in psychiatry and psychology, because of the large impact these two domains can have on society (13, 14). In this context, we believe that questioning the influence of the context on the outcomes of the research, and how to overcome such influence, is crucial if we want to provide more objective and less biased information to our readers.

## 2 Ideology and quantitative research

Many attempts have been developed to try to overcome the issue of confirmation bias in quantitative research and to reduce and/or disclose every element that may impact its conduction and conclusions. One example, on a public health perspective, is the disclosure of financial competing interests. This allows clinicians and researchers to have access to one possible source of bias (i.e. economic). According to the Haute Autorité de Santé in 2023 (15), the French regulatory agency, competing interests are defined as “*a situation in which a person’s links of interest are likely, by their nature or intensity, to raise question on his or her impartiality or independence in the exercise of his or her mission with regard to the case he or she was asked to work on*”. These links of interest may, of course, be financial, but they may also be intellectual. The intellectual form of such link is thus described as a “*benefit in terms of recognition, occasional or regular, in all its forms, notably for the promotion or defense of group interests, such as those of a school of thought, a discipline or a professional specialty*”. To note, the notion of intellectual competing interests is not considered only in France but also worldwide, as it can be found in the British Medical Association (16) and American Psychiatric Association (17) guidelines.

In our opinion, the latter aspect, and especially the promotion or defense of group interests (that will be referred to as “*ideology*” below), is often under-estimated, even though it may have an important impact on research conduction/results. Ideology can be defined as “*a set of ideas, beliefs and attitudes, consciously or unconsciously held, which reflects or shapes understandings or misconceptions of the social and political world*” (18). In terms of psychiatry and psychology, we believe that it can take different forms depending on the level of analysis. One example can be *academic* ideology, which can be defined as the influence of one’s school of thought on the conduction and/or outcomes of research. This example seems particularly relevant in our fields, where many schools of thoughts are at odds, especially when considering psychopathology and psychotherapy. In the latter domains, different schools co-exist (e.g., psychoanalysis, other psychodynamic psychotherapy or cognitive-behavioral therapy), and are sometimes opposed. Thus, it seems legitimate to assume that belonging to one school of thought may influence research conduction in favor of the latter. As an example, in 2008, two authors (19), the first being known for his extensive work on the efficacy of psychodynamic therapies, published a meta-analysis of the effects of long-term psychodynamic therapies, concluding that long-term psychodynamic psychotherapy produced significantly better results in terms of overall efficacy and personality functioning than shorter forms of psychotherapy. This study has been criticized by many authors (e.g., (20, 21)) including cognitive-behavioral therapy pioneer Aaron Beck, and the critical authors even published a re-analysis of the data, concluding on the absence of efficacy (22). In our opinion, this example, among others, illustrates well that, when facing the same data, different school of thought can be associated with opposite conclusions.

But academic ideology is just one example of ideological bias. Any topic associated with a significant polarization of the opinion (either medical, political or sociological (23, 24)) may be subject to this issue. Indeed, given the social utility research can have, and when the research results can lead to significant academical, societal, or political consequences, an important issue emerges: can we really be objective in studying a phenomenon that we are pre-committed to argue for/against it? This question may even raise concerns on the overall objectivity and actual relevance of quantitative approach in our fields, even more when considering that its safeguards such as methodological rigor, peer-reviewing, and the development of ethical guidelines (e.g., the recommendations of the International Committee of Medical Journal Editors (ICMJE, (25)) may be subject to the same type of bias. For example, it has been shown that a reviewer’s political orientation can influence the likelihood of an article containing predominantly socially focused information being published (26) Such critic can also be made on the peer-review process (27, 28). These examples, among others, make us believe that, if these safeguards are significantly helpful, they may be not sufficient to reduce ideological biases in quantitative research.

## 3 Are quantitative approach and scientific method useless in psychiatry?

At this point, one question can be asked: if quantitative approach exists in our fields to overcome human subjectivity, but if it is in fact also prone to the same type of bias than qualitative approach, then what’s its relevance?

Facing this question, we want to warn our readers on the risk of falling into nihilism regarding the utility of scientific method in psychiatry and psychology. Scientific approach has allowed considerable advances, ranging from the development of evidence-based treatments to the development of new understanding of mental health disorders (e.g., (29, 30)). The aforementioned safeguards, if imperfect, allowed to significantly reduce the risk of ideological biases, by allowing: i) the diffraction of the judgment process to different professionals with possibly different ideologies (via peer-reviewing), and ii) the pre- and post-publication analysis of protocols and statistical methodology through pre-registration and raw data diffusion (via the development of open science and ethical guidelines). Moreover, the growing emphasis on reproducibility (i.e., obtaining comparable results using the same data and the same methods, but under different analysis conditions) and replicability (i.e., obtaining comparable results in all studies designed to answer the same scientific question, each having obtained its own data but using the same methods) may also help to detect biased studies and to protect from long-term duration of ideological biases. Altogether, our aim here is not to dismiss quantitative approach, but more to contribute to ongoing debates regarding its improvement. Indeed, we believe that considering psychiatric quantitative science purely objective and free of biases is as problematic as considering it totally useless and subjective. Each approach has its own pitfalls, and qualitative approach shares many of them in terms of ideological influence (e.g., regarding socio-political ideologies and psychoanalysis in the French context (31)). We believe it’s our role as researchers to shed light on the limitations of our practice to enhance the readers’ trust towards our results.

Thus, to move into this direction, we believe that quantitative research can learn from the rapidly expanding field of qualitative research, especially on how its researchers consider the risk of impact of preconceptions on their results. Indeed, the methodology requires authors to write a logbook describing why they became interested in a subject, what results they expect from their study, and what might surprise or challenge them (32). Accessible alongside the publication of their results, readers can thus make their own opinion on the risk of confirmation bias. This makes it possible, without making it disappear, to significantly reduce it, or at least make it visible. This kind of prior declaration seems central to implement in quantitative research, especially in fields with potential societal or political impact. Indeed, when looking at the definition of intellectual link of interest we presented earlier (“*benefits in terms of recognition [ … ] notably for the promotion or defense of group interests”)*, and as it is mandatory to declare financial competing interests, it would seem logical that a declaration of ideological competing interest (whether academic, sociological, political or other) should be at least considered. Indeed, we believe that a fully transparent position should include a disclosure on from *where* the researcher is talking, as the latter may influence the objectivity of the results.

## 4 Reflections on ideological competing interests’ disclosure implementation challenges

We are aware that this proposition is provocative and may be difficult to implement in real life. Researchers have necessarily their own opinions, and the boundary is subtle between ideology and personal theoretical or political opinions. Thus, many practical challenges can be associated with the implementation of such a policy.

For example, there may be a risk of stigmatization of certain viewpoints leading to discouragement of researchers from exploring controversial topics. Indeed, if working on a “hot” and polarizing topic may include personal opinions’ disclosure, researchers having one opinion but evolving in a context favorable to another may start to withdraw from this specific topic exploration to not suffer any consequences (like rejection from social or academic spheres), which could in turn enhances the risk of publication bias (with only research matching the context’s opinion being conducted). However, this risk could be decreased by restricting the content of this disclosure only to the researchers’ preconceived opinions on the actual subject of the study, without compelling them to fully describe all their personal opinions outside of the research subject.

Also, there may be a risk of increasing the actual global mistrust in psychiatric science, both from public and practitioners [e.g., (33)]. Indeed, if ideological interests are disclosed, and if the results found are in line with these interests, readers may be prone to over conclude that these results are inherently biased, especially when considering that these readers may also be prone to confirmation bias, and when considering the overall low level of knowledge on how research is conducted and statistical analyses work (34, 35). If this risk should be carefully considered, we also believe that this mistrust partly relies on the actual non-disclosure of this type of competing interests that have long been the subject of concerns, and providing a way to address these concerns may thus be a way to enhance the readers’ trust.

Finally, given that the present paper can be seen as a practical example of confirmation bias and ideology, we wanted to lay the foundation stone of ideological competing interests’ disclosure. Indeed, the four authors have their own opinions regarding these topics, and notably on the risk of influence of ideologies on the conduction and production of science. Thus, the examples and the references we chose can be seen as subjective, partials, and selected to support our preconceived opinions. We described the latter in the Author’s Note section. Overall, we hope that this article will contribute to pursue the reflection on this important topic.

## Author’s Note

Given our proposition to disclose ideological competing interests, we wanted to disclose our own regarding this paper. MB is a psychiatrist and psychotherapist specialized in the study and treatment of patients suffering from personality disorders. He received his MD in psychiatry in Lyon (France), a city where large academic conflicts took place in the last decades between psychoanalysts and biological psychiatrists, leading to important ideological consequences in terms of psychiatry training. His preconceived opinions are that quantitative *and* qualitative approaches can both be useful and should be considered, from psychoanalysis to biological psychiatry, notably because they all allowed great progress in the understanding and treatment of patients with personality disorders. Finally, he also has a great interest in the study of the link between society organization and psychiatry, especially regarding the development of personality disorders.

CG completed an engineering degree in computational sciences and a PhD in philosophy of medicine. As an adolescent psychiatrist who follows the French university curriculum quite classically, he is certainly interested in the discussions raised by such opinion articles at the border of methodology, philosophy and science studies, but he is undoubtedly and unconsciously moved by the implicit argument that such an article, in an indexed journal, brings him “points” for his institutional recognition by his peers.

PE is a child psychiatrist with a clinical practice in the field of psychotrauma and victimology. Before her practice of psychiatry, she completed a medicine-science curriculum and a master’s degree in neuroscience. She then started her training in psychiatry through institutional psychotherapy and psychoanalytic culture in Reims. She completed her internship in Lyon, where the neurobiological approach was predominant in academic teaching. She now tries to have a clinical practice as integrative as possible, with a growing interest in the socio-political aspects of psychotrauma. Finally, she belongs to scientific societies of the discipline (SFPEADA and API), and the union of hospital psychiatrists (SPH).

Finally, BF is a professor of biostatistics but is ambivalent about the dominant place of his discipline in the epistemology of contemporary biomedical research. As a child psychiatrist, he is critical of the dominant position of neuroscientific thought in this field and is interested in the human sciences and in psychoanalysis. He regrets the latter’s lack of openness to the experimental approach.

## Author contributions

MB: Writing – review & editing, Writing – original draft. CG: Writing – review & editing, Supervision. PE: Writing – review & editing. BF: Writing – review & editing, Supervision.
